# Secreted aspartyl proteinase (PbSap) contributes to the virulence of *Paracoccidioides brasiliensis* infection

**DOI:** 10.1371/journal.pntd.0006806

**Published:** 2018-09-27

**Authors:** Daniele Gonçalves Castilho, Alison Felipe Alencar Chaves, Marina Valente Navarro, Palloma Mendes Conceição, Karen Spadari Ferreira, Luiz Severino da Silva, Patricia Xander, Wagner Luiz Batista

**Affiliations:** 1 Department of Microbiology, Immunology and Parasitology, Universidade Federal de São Paulo, São Paulo, SP, Brazil; 2 Department of Pharmaceutical Sciences, Instituto de Ciências Ambientais, Químicas e Farmacêuticas, Universidade Federal de São Paulo, Diadema, SP, Brazil; Rutgers University, UNITED STATES

## Abstract

Paracoccidioidomycosis (PCM) is the most prevalent deep mycosis in Latin America and is caused by fungi from the *Paracoccidioides* genus. Virulence factors are important fungal characteristics that support the development of disease. Aspartyl proteases (Saps) are virulence factors in many human fungal pathogens that play an important role in the host invasion process. We report here that immunization with recombinant Sap from *Paracoccidioides brasiliensis* (rPbSap) imparted a protective effect in an experimental PCM model. The rPbSap-immunized mice had decreased fungal loads, and their lung parenchyma were notably preserved. An aspartyl protease inhibitor (pepstatin A) significantly decreased pulmonary injury and reduced fungal loads in the lung. Additionally, we observed that pepstatin A enhanced the fungicidal and phagocytic profile of macrophages against *P*. *brasiliensis*. Furthermore, *PbSAP* expression was highly altered by environmental conditions, including thermal stress, dimorphism switching and low pH. Hence, our data suggest that PbSap is an important virulence regulator in *P*. *brasiliensis*.

## Author summary

Paracoccidioidomycosis is a systemic fungal disease occurring in Latin America and more prevalent in South America. The disease is caused by the dimorphic fungus *Paracoccidioides* spp., its pathogenesis is multifactorial and few virulence factors have been recognized in this fungus. Some virulence factors, like the secreted aspartic proteases (Saps), have been described playing an important role in adhesion, invasion and tissue damage in many pathogenic microorganisms. Thus, the inhibition of one of the many infection stages may contribute to the containment of the pathogen and thus should help in the treatment of disease. We have here produced an aspartyl protease recombinant of *Paracoccidioides* (rPbSap) and shown that rPbSap-immunized mice decreased disease progression. Besides that, when infected mice were treated with an aspartyl protease inhibitor we also observed a significant reduction of fungal infection. In addition, PbSap expression was modulated by different stress conditions, especially in low pH environment. These findings provide insights for the design of new antifungal therapies for this important fungal disease.

## Introduction

The pathogenic fungi *Paracoccidioides brasiliensis* and *Paracoccidioides lutzii* are the etiological agents responsible for paracoccidioidomycosis (PCM). PCM is geographically limited to Latin America; Brazil has the highest number of reported cases [1,2]. *Paracoccidioides* is a thermally dimorphic fungus that exists in a mycelial phase at 25°C or ambient temperature and, when grown at 37°C, appears as yeast. *P*. *brasiliensis* infection occurs after the inhalation of conidia produced during the mycelial phase, which triggers differentiation into pathogenic yeast cells in the lungs. The development of disease depends both on factors associated with the host immune response and on the characteristics of the infectious agent, especially its virulence. However, few molecules have been effectively characterized as virulence factors in *P*. *brasiliensis* [3].

The extracellular proteases of pathogenic fungi perform important functions during infection; for example, some hydrolytic enzymes promote adhesion and tissue invasion by hydrolyzing proteins in host cell membranes [4]. The activity of proteinases and phospholipases is directly related to the establishment of infection [4–6]. Hydrolytic enzymes produced by *Candida albicans* represent the greatest factors that have been associated with virulence [7]. Aspartyl proteases are a family of proteolytic enzymes that play an important role in host invasion in many pathogenic fungi. *C*. *albicans* exploits many virulence factors to infect the host. The most important is a family of ten secreted aspartic proteases (Saps) that cleave numerous peptides and proteins, often deregulating the host's biochemical homeostasis [4,6]. Saps are also known to be immunogenic and to induce protective host defense in animal models [8,9]. The aspartyl protease of *Aspergillus fumigatus*, aspergilopepsin, is secreted in large quantities during lung infection in an animal model [10]. An aspartyl protease associated with the cell wall was detected in *Coccidioides posadasii*, and the recombinant protein was reported to be a putative candidate for a new vaccine [11].

In *Paracoccidioides lutzii*, a secreted aspartyl protease (PbSap) with highest identity to the aspartyl protease of *C*. *posadasii* (88%), followed by *A*. *clavatus* (87%) and *A*. *terreus* (87%), was identified and characterized. The similarity of PbSap to the Saps of *C*. *albicans* ranges from 40–47% [12]. In addition, analysis of the transcriptome of *P*. *lutzii* during its transition from mycelium (infecting form) to yeast (pathogenic form) revealed that the PbSap transcript is up-regulated in the yeast phase of the fungus [13]. Recently, analysis using real-time qPCR during biofilm formation by *P*. *brasiliensis* revealed an increase in the expression of aspartyl proteinase genes; these profiles are potentially associated with virulence [14].

Quantitative proteomic analyses using the same *P*. *brasiliensis* isolate (Pb18) with different degrees of virulence showed significant differences in the protein content between isolates, and the proteins found to be differentially expressed in the virulent isolates were presented as potential virulence factors [15]. Among the proteins with increased expression in the virulent isolate was the vacuolar A protein, also known as aspartyl protease (PbSap). The expression of this protein was 8-fold higher in virulent Pb18 (vPb18) than in the attenuated Pb18 (aPb18) isolate. This result was validated by real-time PCR. In addition, the expression of PbSap was increased in an aPb18 isolate after two consecutive animal passages [15]. In the present study, we produced and purified recombinant PbSap (rPbSap), which was recognized using serum from PCM patients. Immunization with rPbSap led to a reduction in fungal load in an experimental PCM model. In addition, the expression of PbSap can be modulated during dimorphism or with pH changes. Finally, the effect of pepstatin A, an aspartyl protease inhibitor in *C*. *albicans*, was investigated in the PCM model. After exposure to the proteinase inhibitor, fungal loads in the lungs were significantly reduced. These inhibitory effects caused by pepstatin A application reduced the virulence phenotype of experimental PCM, indicating the usefulness of the protease inhibitor as a potential antifungal agent in PCM.

## Materials and methods

### Fungal isolate and growth conditions

*P*. *brasiliensis* isolate Pb18 was used in all experiments. Yeast extract-peptone-dextrose modified medium (mYPD) [0.5% yeast extract, 1% casein peptone, and 0.5% glucose, pH 6.5] was used to cultivate yeast or mycelium cells, which were cultured at 37°C or 25°C, respectively. To induce protease secretion, *P*. *brasiliensis* yeast cells were also cultured in mYPD liquid supplemented with 1% bovine serum albumin (BSA).

### Animal use and ethics statement

Male BALB/c mice (6 to 8-weeks-old) were maintained under specific pathogen-free conditions at a temperature of 23–24°C with a light/dark cycle of 12 h and were provided with food and water *ad libitum*. Animal experimentation was approved by the Ethics Committee on the use of animals at the Federal University of São Paulo (CEP 1631220814). Animals were handled according to the Brazilian National Council for Animal Experimentation Control (CONCEA) guidelines.

### RNA extraction and qRT-PCR

*PbSAP* transcripts from *P*. *brasiliensis* grown under different experimental conditions were quantified by RT-qPCR. Total RNA was obtained from Pb18 yeast cells using the TRIzol reagent (Thermo Fisher Scientific, Bremen, GA, USA) after oxidative, osmotic and thermal stress conditions and during M-Y and Y-M transitions, as described previously [16–18]. Next, cDNA was synthesized with RevertAid Premium Reverse Transcriptase (Thermo Fisher Scientific, Bremen, GA, USA) according to the manufacturer's instructions. The cDNA preparations were used in qRT-PCR reactions to measure the expression levels of *PbSAP*. Reactions were performed using the SYBR Green/ROX qPCR Master Mix 2X (Thermo Fisher Scientific, Bremen, GA, USA) according to the manufacturer's instructions. Briefly, a 10-μL total volume was used for each PCR reaction, which consisted of 1× SYBR GreenPCR Master Mix, 250 nmol of the reverse primer, 250 nmol of the forward primer and 2 μL of cDNA. The cycling parameters were 50°C for 10 min, 95°C for 5 min and 40 cycles of 95°C for 30 s and 60°C for 1 min. A non-template control was used to detect any contamination. The quality of the reactions was determined from the dissociation curves. The results obtained were analyzed for baseline and threshold cycle values (Ct) using Step OnePlus software (Thermo Fisher Scientific, Bremen, GA, USA). The relative expression ratio (experimental/control) was determined based on the 2^-ΔΔCT^ method [19] after normalization to the transcript levels of α-tubulin (*α-TUB*) and 18S ribosomal RNA (*18S*). DNA contamination was evaluated via PCR amplification of the GP43 gene (Accession No. U26160). Negative controls contained neither DNA nor RNA. The nucleotide sequences of the forward and reverse primers were as follows: *GP43* 5’-TGTCACCCTTTTGCCAGT TG-3’ and 5’-TTCCCAAAACGGCTTCGA-3’; *PbSAP*, 5’-GATGACTCTGAGGCTACCTTTG-3´ and 5´-ATCGAGATCAACCTCCCAGTA-3’; α-*TUB*, 5’-CGGCATATGGAAATACATGGC-3’ and 5’ GTCTTGGCCTTGAGAGATGCA 3’; and *18S*, 5'-CGGAGAGAGGGAGCCTGAGAA-3' and 5’-GGGATTGGGTAATTTGCGC-3’. Experiments were performed in biological triplicate.

#### Cloning of *PbSAP* cDNA, expression of PbSap in bacteria, recombinant protein purification and antibody production

Initially, primers were designed to amplify the 1,203-bp cDNA of *PbSAP* (PADG_00634). The nucleotide sequences of the forward and reverse primers were as follows: 5´-ACC*GAATTC*TATGAAGTTCTCTCTG-3´ and 5´-ACC*CTCGAG*TCACTGTCTAGCCTTCG-3´, which contained *Eco*RI and *Xho*I restriction sites (underlined), respectively. The PCR product was cloned into the pJET 1.2/blunt vector (Thermo Fisher Scientific, Bremen, GA, USA) and then subcloned into the pET28a expression vector. Correct in-frame ligation was verified by endonuclease restriction and sequencing. The recombinant plasmid was transformed into *E*. *coli* BL21/pLysS (Novagen, Madison, WI, USA) using the heat-shock method [20]. For purification of recombinant PbSap (rPbSap), individual bacterial clones were cultivated to log phase (A_600_ = 0.6) at 37°C with shaking in Luria-Bertani (LB) medium containing 30 μg/mL of kanamycin. Isopropyl-β-D-thiogalactopyranoside (IPTG) was added to the growing culture to a final concentration of 1 mM to induce protein expression for 3 h. Bacterial cell pellets were harvested by centrifugation at 10,000 *g* for 5 min, resuspended in buffer [50 mM Tris-HCl (pH 7.0), 0.2 M NaCl, 10% sucrose and 1 mM phenylmethylsulfonyl fluoride] and disrupted by three 5-min rounds of sonication. Precipitates were obtained by centrifugation at 10,000 *g* for 5 min and resuspended in buffer containing 100 mM NaH_2_PO_4_, 10 mM Tris-HCl (pH 8.0) and 8 M urea. The supernatant was chromatographed using Ni-nitrilotriacetic acid (NTA) columns (QIAGEN, Maryland, USA) according to the manufacturer’s instructions. The rPbSap fractions eluted at pH 4.5 were pooled for further use. Two hundred micrograms of purified rPbSap were electrophoresed using 12% SDS-PAGE [21] gels that were then stained with Coomassie brilliant blue.

rPbSap refolding was done with chaotropic agents’ concentration gradient dialysis. The solution of denatured protein (0.2 mg/mL) was dialyzed against 2 L of freshly made 6, 4, 2, 1, 0.5, and 0 M urea, respectively, with 5 mM Tris (pH 7.4). With each concentration, the protein was dialyzed 12 h at 4°C. Groups of mice BALB/c were injected i.p. with 0.2 mL of a 1:1 mixture of heat aggregated rPbSap (100 μg) in Imject Alum Adjuvant (ThermoFisher). The mice were boosted 3 times at one-week intervals with the same amount of rPbSap via the same procedure. Serum containing anti-rPbSap polyclonal antibodies was tested and aliquoted at -20°C. Mice were also bled before immunization to obtain preimmune sera.

### Immunoblotting assays

Western blot analysis was performed with sera from hyperimmune mice or patients with PCM. Purified rPbSap was electrophoresed on 12% SDS-PAGE gels and electroblotted to nylon membranes that were blocked with 5% (w/v) non-fat dried milk in 1x TBS-T for 1 h at room temperature with shaking. Aspartyl protease was detected using sera from mice containing anti-rPbSap polyclonal antibodies (produced in this work) or sera from PCM patients. After incubation with the appropriate HRP-conjugated secondary antibodies anti-mouse IgG (whole molecule) (at 1:2,000 dilutions, KPL), the blots were developed using the Super Signal system (Thermo-Pierce–Rockford, IL). Image acquisition and densitometry were performed using a chemiluminescence documentation system (UVITEC, Cambridge, UK). Negative controls included preimmune sera from mice or sera from healthy patients. Total protein extracts from yeast were obtained as described [22] and subjected to western blot analysis using the anti-rPbSap polyclonal antibody to detect PbSap in total protein extracts from Pb18 yeast cells.

### Effects of pH on the expression of PbSap

*P*. *brasiliensis* yeast cells were cultured in mYPD liquid ranging from pH 3–6.5 using BSA as a substrate at 37°C for 7 days with shaking. Total protein extracts were obtained as described previously and electrophoresed on 12% SDS-PAGE gels that were then stained with Coomassie brilliant blue. Aspartyl protease was detected using the anti-rPbSap polyclonal antibody.

### Peptide mass mapping by MALDI-TOF-MS/MS

*P*. *brasiliensis* yeast cells were cultured in mYPD liquid using BSA as a substrate at 37°C for 7 days in low pH (pH 4.0). Total protein extracts were obtained and electrophoresed on 12% SDS-PAGE. The 66 kDa and 44 KDa lines were cut into 5 slices and slices from identical gel areas were combined. The in-gel digestion was carried out according to Stenballe and Jensen [23]. Protein digestion was identified by mass spectrometry on a Bruker MALDI-TOF instrument, according to Gyndry et al., [24].

### Immunolocalization of PbSap

*P*. *brasiliensis* yeast cells were cultured in mYPD until they reached exponential growth, and 1x10^6^ yeast cells were fixed for 30 min at room temperature in ice-cold methanol (Merck) and blocked overnight at 4°C in 1x PBS solution containing 3% BSA. The yeast cells were incubated with the anti-rPbSap polyclonal antibody at a 1:100 dilution for 4 h at room temperature, followed by incubation with the conjugate antibody (Anti-Mouse IgG (whole molecule)-FITC antibody, Sigma Aldrich, St. Louis, MO, USA) at a 1:100 dilution for 2 h at room temperature. The cells were co-stained with 5 mM Calcofluor white to visualize their cellular morphology. Microscopy slides were mounted with a small aliquot of the preparations in antifading Vectashield (Vector Laboratories, Burlingame, CA, USA) and sealed. The double labeling was analyzed by confocal microscopy (Carl Zeiss LSM-510 NLO, Germany), and multiple images (at least 5–6 slices of Z = 0.45 μm) were captured. Image acquisition and comparative analyses of intensity were performed under similar instrument adjustment parameters.

### Immunization and intratracheal infection of BALB/c mice

BALB/c mice were immunized with 100 μg of purified rPbSap by intraperitoneal injection as previously described. Imject Alum adjuvant (Thermo Fisher) was used as an adjuvant. Three groups of mice (n = 6 animals each) were used. Two controls were included: non-immunized mice and mice injected with adjuvant without the recombinant protein. After immunization, the mice were challenged i.t. with 1x10^5^ mYPD-grown virulent yeast cells (vPb18) [15] in 40 μL of saline solution. Briefly, the mice were anesthetized, their trachea was exposed and injected with 1x10^5^ viable vPb18 yeast cells. The incisions were sutured with 4–0 silk. After 30 days of infection, the lungs were excised, and the numbers of viable microorganisms in the lungs were determined by enumerating CFUs [25]. Fragments of lungs were fixed in 10% buffered formalin (Merck, Germany) for 24 h and subjected to histopathological analysis via hematoxylin and eosin (H&E) staining. IFN-γ production was analyzed in lung tissue using quantitative enzyme-linked immunosorbent assay (ELISA). These experiments were performed four times.

### Effect of PbSap inhibition in experimental PCM

Male BALB/c mice were infected i.t. with 1x10^5^ viable vPb18 yeast cells as previously described, and after 30 days of infection, the animals were treated 4 times, once a week, with 0.06 mg/kg of pepstatin A (Sigma Aldrich, St. Louis, MO, USA) in 200 μL of saline solution or with 10 mg/kg of itraconazole (ITR) (Sigma Aldrich, St. Louis, MO, USA) [26] via intraperitoneal injection. BALB/c mice were divided into three groups (n = 6 animals per group) as follows: 1) Control (untreated infected mice); 2) Infected mice treated with pepstatin A and 3) Infected mice treated with ITR. After 30 days of infection, all animals were euthanized, CFUs in the lungs were determined and histopathological analysis (H&E staining) was also performed.

### Phagocytosis assay and antifungal activity of macrophages

*In vitro* phagocytosis experiments were performed with the THP1 cell line. Phagocytic tests were performed according to Parente-Rocha et al. [27] with minor modification. THP1 cells (10^6^) were plated in 24-well tissue culture plates (TPP, Switzerland) with one coverglass per well in RPMI medium (Gibco, Gaithersburg, MD, USA) with 10% fetal bovine serum and then incubated for 24 h at 37°C and 5% CO_2_ for adherence. After 24 h, adherent THP1 cells were differentiated into macrophages using 50 ng/mL phorbol 12-myristate 13-acetate (PMA) (Sigma Aldrich, St. Louis, MO, USA) in RPMI medium for 24 h at 37°C and 5% CO_2_. After 24 h, the macrophages were stimulated with 2 ng/mL recombinant murine IFN-γ (BD Biosciences, San Jose, CA, USA) and reincubated at 37°C and 5% CO_2_ overnight. Then, 10^6^ yeast cells of *P*. *brasiliensis* were treated with pepstatin A at 15 or 30 μM for 1 h at 37°C in RPMI medium. Cells were recovered by centrifugation at 10,000 g for 5 min and added to the macrophages at a yeast:macrophage cell ratio of 10:1, followed by incubation at 37°C and 5% CO_2_ for 48 h in fresh RPMI medium containing 20 ng/mL IFN-γ. The coverglasses were stained with a 1/20 solution of Giemsa (Sigma Aldrich, St. Louis, MO, USA), and the e phagocytic index was determined according to Popi et al. [28].

To assess the effect of pepstatin A on the antifungal activity of macrophages, *P*. *brasiliensis* yeast cells were treated with pepstatin A at 15 or 30 μM for 1 h at 37°C. Cells were recovered by centrifugation at 10,000 g for 5 min and added to the macrophages at a yeast:macrophage cell ratio of 10:1, followed by incubation at 37°C and 5% CO_2_ for 48 h in fresh RPMI medium containing 20 ng/mL IFN-γ. The numbers of *P*. *brasiliensis* yeast cells were determined by enumerating CFUs. After interacting, the macrophages were lysed by the addition of cold sterile water and plated on BHI agar supplemented with 4% fetal bovine serum and 5% spent culture medium of Pb192. CFUs were enumerated after 7 days of incubation at 37°C.

### Statistical analysis

Data are expressed as the means ± SD. Significance was assessed by one-way analysis of variance, with Student’s *t*-tests used for comparisons. Results with *P*<0.05 were considered statistically significant.

## Results

### Characterization of *P*. *brasiliensis PbSAP* (Pb18 strain)

The *PbSAP* coding sequence from the Pb18 strain was 1,460 bp long, consistent with the predicted sequence (UniProt accession number PADG_00634), and it contained an open reading frame of 1,200 bp. Tacco and co-workers [12] identified and characterized an aspartyl protease in the Pb01 strain of *P*. *brasiliensis* (currently called *Paracoccidioides lutzii*). This protein has 92% identity to PbSap from the Pb18 strain, and both aspartyl proteases share important attributes that are essential for *SAP* function. The similarity of *PbSAP* to the *C*. *immitis* and *C*. *posadassi SAPs* was 76%, followed by a similarity of 73% to the *A*. *clavatus*, *A*. *terreus* and *Histoplasma capsulatum SAPs*. *PbSAP* showed identities to the *C*. *albicans SAPs* 1–10 ranging from 24–30%, with high similarity to *SAP8*.

### Gene expression analysis of *PbSAP* under different stress conditions

**Fig 1:**
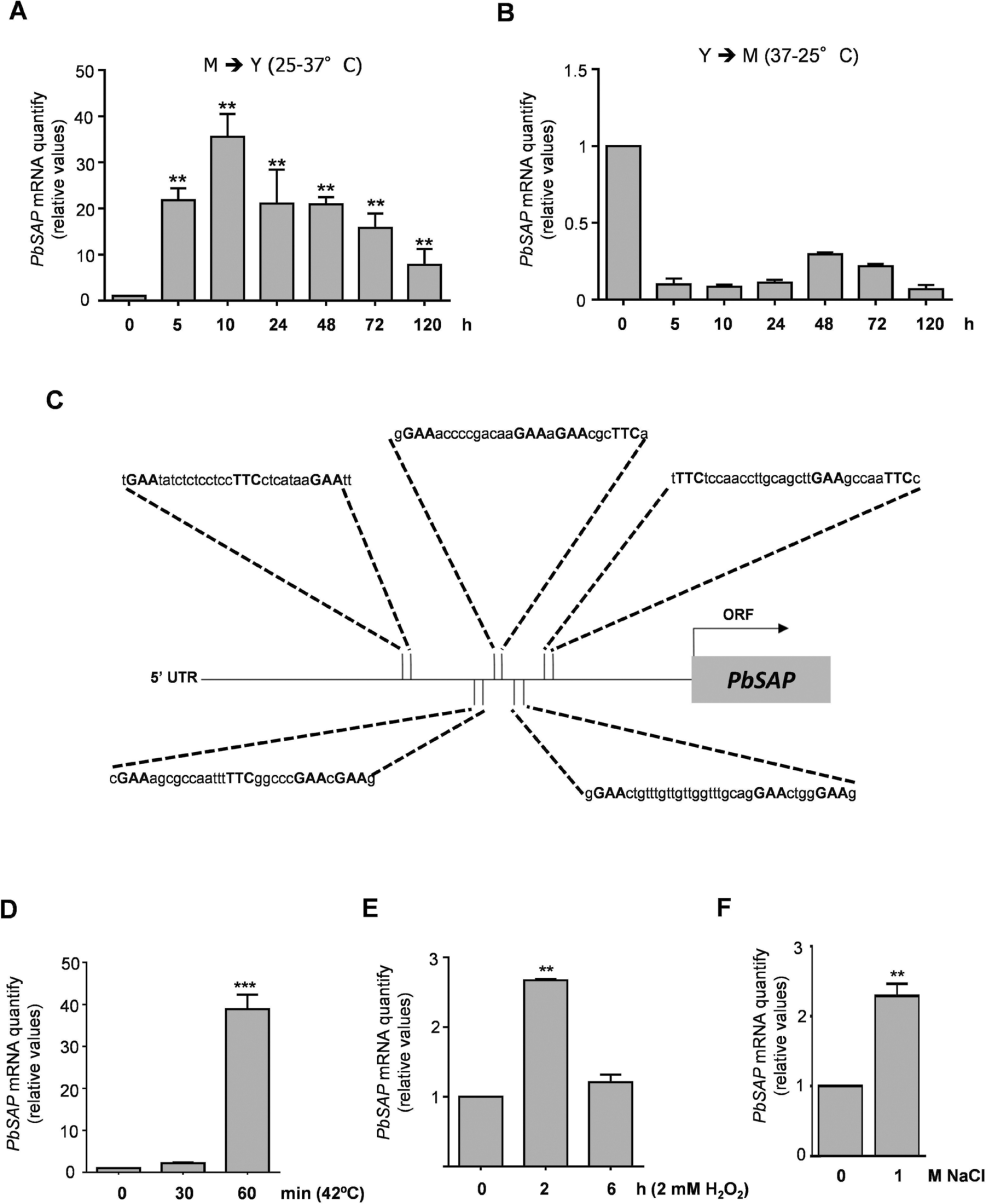
Reverse transcriptase qPCR of *PbSAP* from *P*. *brasiliensis* under multiple conditions and schematic representation of the *PbSAP* 5’ UTR region. *PbSAP* (PADG_00634) transcript levels were measured during the mycelium to yeast (M-Y) (A) or yeast to mycelium (Y-M) transition (B). Schematic representation showing the putative non-conventional heat shock elements (ncHSE) motifs in the promoter region of *PbSAP* (C). Quantitative real time RT-PCR of *PbSAP* from Pb18 yeast cells after heat shock at 42°C for 30 or 60 min (D), treatment with 2 mM H_2_O_2_ at 2 or 6 h (E) and different NaCl concentrations (F), as indicated. The change in transcriptional levels was calculated via the 2^-ΔΔCt^ method, with two housekeeping genes (*α-TUB* and *18S*). All of the data shown in this figure were analyzed using Student’s t-test. Error bars correspond to the standard deviation of measurements performed in triplicate, and asterisks indicate statistically significant differences in expression (*p < 0.05, **p<0.01 and ***p<0.001).

Gene expression of *PbSAP* was assessed during dimorphic switching and oxidative, osmotic and thermal stress. Temperature controls the dimorphic transition of *P*. *brasiliensis*, and pathogenicity is intimately linked to dimorphic switching [16]. In the mycelium-to-yeast transition (M-Y transition), the transcript levels of *PbSAP* were up-regulated during the early stage (10 h—31.6-fold), and they remained elevated after the complete transition (120 h– 9.5-fold) (**Fig 1A**). Conversely, during the yeast-to-mycelium transition (Y-M transition), we noticed down-regulated transcript levels of *PbSAP* (**Fig 1B**). The most potent virulence factors are up-regulated during the M-Y transition [29], thus providing early evidence that *PbSAP* is a possible virulence regulator. In addition, the *PbSAP* gene showed an mRNA expression pattern similar to those of the HSP70, HSP82, and HSP104 genes in both transitions, as observed by Goldman and co-workers (2003) [30]. Therefore, we investigated the 5' UTR region of *PbSAP* for the presence of putative heat-shock elements (HSE). The target genes of the heat-shock transcription factor (HSF) contain a cis-acting sequence, the HSE, which consists of multiple inverted repeats of the sequence 5´-nGAAn-3´. We did not detect obvious HSE (classical HSE, cHSE) in the *PbSAP* promoter region. However, the promoter region of *PbSAP* contained continuous inverted repeats of pentanucleotide units with sequences that diverged from cHSE (**Fig 1C**). We detected five putative non-classical HSE (ncHSE) motifs (**Fig 1C**). These putative ncHSE motifs contain different gap lengths between the units (nGAAn) that range from 3 to 15 bp. Thus, we investigated whether the *PbSAP* gene is heat-inducible. The expression of *PbSAP* was 40-fold greater in response to thermal stress at 42°C for 1 h than in the control yeast at 37°C (**Fig 1D**). These data suggest that the PbSap protein is heat-inducible, despite its lack of cHSE. We also analyzed *PbSAP* expression after oxidative and osmotic stress. The transcript levels of *PbSAP* increased in the early stages (2 h, 3-fold) of oxidative stress (**Fig 1E**) and osmotic (3-fold) stress (**Fig 1F**).

### PbSap expression in *E*. *coli* and polyclonal antibody production

**Fig 2:**
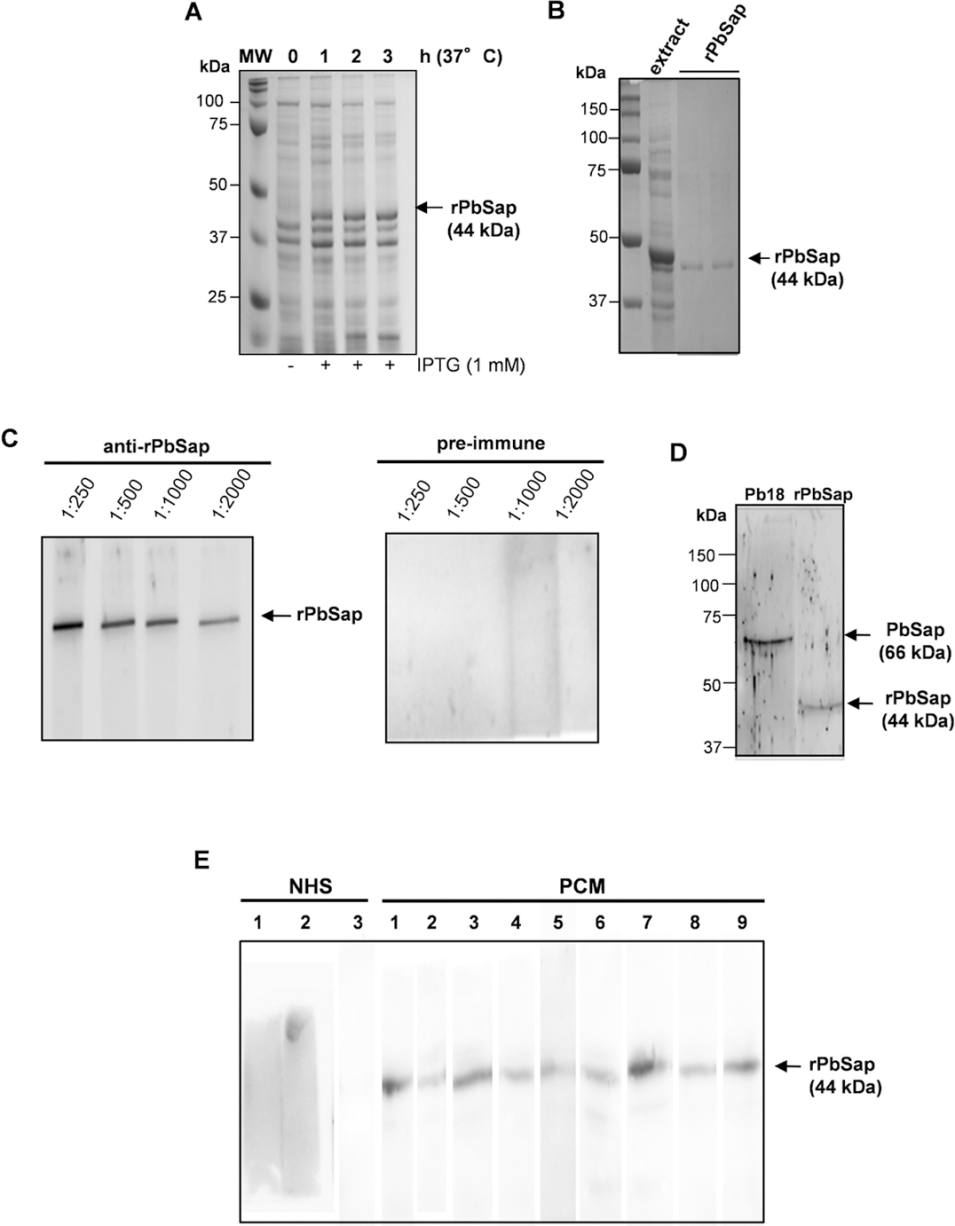
SDS-PAGE and western blot analysis of the recombinant PbSap. Total bacterial extracts from recombinant bacteria expressing rPbSap (A) and the respective protein eluted from Ni-NTA columns with pH 4.5 buffer (B). Western blot analysis of mouse immune sera showing the production of anti-rPbSap (C) and analysis using the total protein extract (25 μg) of *P*. *brasiliensis* yeast (D). Immunoblot reactivity of PCM patient’s sera with rPbSap. Controls were serum from a healthy individual (NHS) and human sera were tested at a 1:200 dilution (E).

In *E*. *coli*, His-tagged PbSap (400 amino acids) was expressed as a major insoluble cytoplasmic protein. A molecular mass of approximately 44.65 kDa was calculated for rPbSap, including its vector sequence, and its observed SDS-PAGE mobility was compatible with this value (**Fig 2A**). Lysis of the bacterial cells was followed by purification of rPbSap using a 6x Histidine-tag/Ni-NTA system (**Fig 2B**). rPbSap protein was refolding and used to immunize mice. Anti-rPbSap mouse immune sera recognized rPbSap at titers up to 1/2,000 when tested with 200 ng of recombinant protein (**Fig 2C**). No reactions were detected with mice preimmune sera. Using anti-rPbSap antibodies with total protein extracts from Pb18, we noticed only a single band with a molecular mass of 66 kDa, which corresponds to the glycosylated form of PbSap (**Fig 2D**). In *P*. *brasiliensis*, the PbSap deduced amino acid sequence revealed two N-glycosylation sites predicted at positions 139–142 and 339–342, the same N-glycosylation sites predicted to aspartyl protease of *P*. *lutzii*. We performed an assay using endoglycosidase H to confirm that PbSap of *P*. *brasiliensis* is also glycosylated. After treatment of the total protein extract from *P*. *brasiliensis* yeast cells with endoglycosidase H a protein with molecular weight of 44 kDa was recognized by polyclonal antibodies anti-rPbSap (**S1 Fig**). These data support the important information that 66 kDa protein is the glycosylated form of PbSap in *P*. *brasiliensis*. According to the Clustal Omega algorithm (http://www.ebi.ac.uk/Tools/msa/clustalo/), the PbSap sequence has a 13-amino-acid region (LLAATTTLLGTSSA) that contains T cell epitope characteristics. In addition, we also evaluated the immunogenicity of PbSap using western blot analysis with PCM patients’ sera. When rPbSap was used in a western blot assay to detect specific antibodies in sera from ten PCM patients, we observed that all the sera recognized rPbSap (**Fig 2E**). No reactions were detected with sera from healthy individuals (**Fig 2E**). The recombinant protein rPbSap was not recognized by sera from patients with candidiasis, histoplasmosis and aspergillosis. These data suggest that PbSap can stimulate a specific immune response and act as an antigen of *P*. *brasiliensis*.

### Expression of PbSap at low pH values

**Fig 3:**
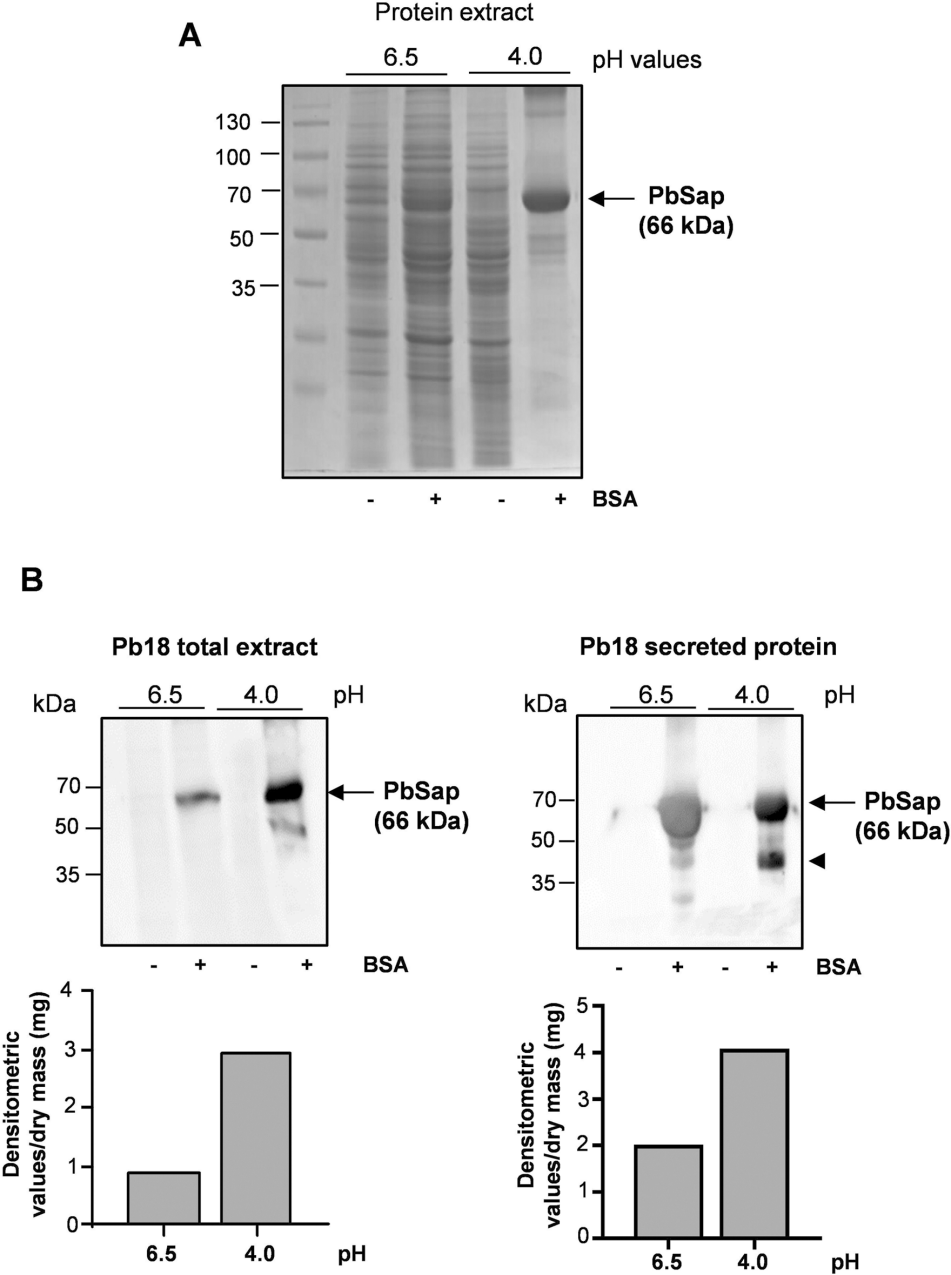
SDS-PAGE, western blot and RT-PCR analysis of PbSap in *P*. *brasiliensis* yeast cells cultured in low pH. Profile of the Coomassie brilliant blue stained gel (12% SDS-PAGE) using the total protein extract (25 μg) of *P*. *brasiliensis* yeast cultured in pH 6.5 or pH 4.0 with or without BSA. (A). Immunobloting analysis of intracellular and secreted proteins in low pH (B). Relative densitometric values of bands were normalized by dry mass and the results are shown in the bar graphs. the arrowhead indicates the non-glycosylated form of PbSap (44 kDa).

Aspartyl proteases generally act at low pH [4]. To assess protease production, *P*. *brasiliensis* yeast cells were grown over a pH range using BSA to induce protease secretion. PbSap expression increased considerably at low pH (4.0) (**Fig 3A**). Western blot analysis using the polyclonal anti-rPbSap antibody showed that intracellular and secreted PbSap expression was substantially higher at low pH (**Fig 3B**). Interestingly, under these growth conditions, the Pb18 exhibited slow growth in acidic pH. In Fig 3B, the polyclonal antibodies recognized two bands with distinct size, 66 kDa and 44 kDa. We supposed that polyclonal antibody recognized PbSap in the glycosilation form (66 kDa) and no-glycosilaton form (44 kDa). Mass spectrometry analysis was performed to confirm this hypothesis. Initially, the bands corresponding to 66 kDa and 44 kDa were excised from the Comassie-stained 1D gel and digested with trypsin. Protein digestion were identified by mass spectrometry by MALDI-TOF-MS/MS. Peptides mass fingerprints obtained in both bands were similar to the prediction fragmentation profile when PbSap was digested using trypsin.

**Fig 4:**
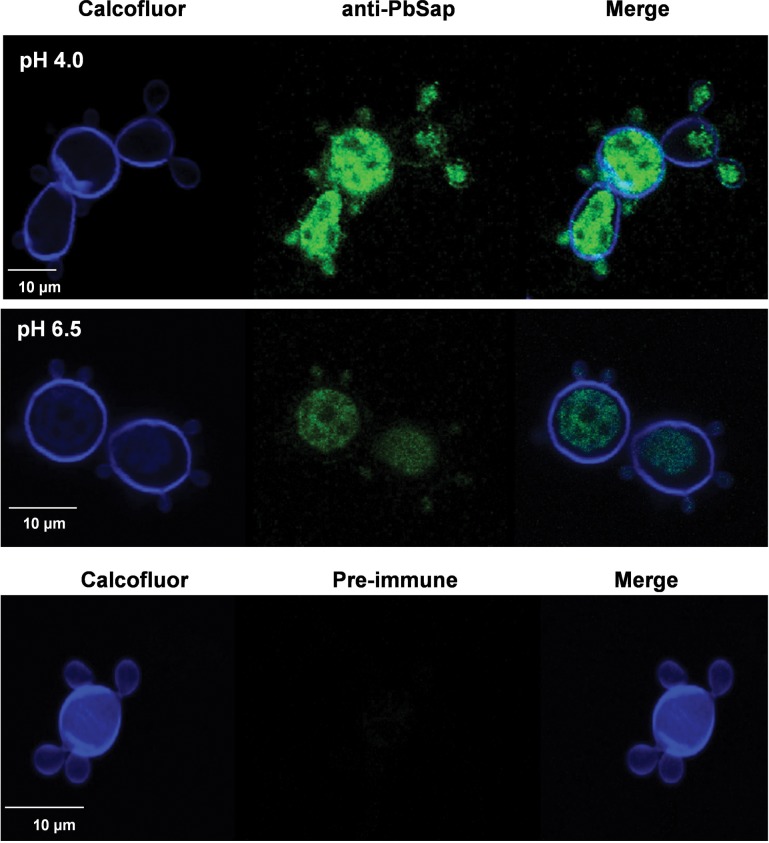
Immunofluorescence assay of PbSap in *P*. *brasiliensis* yeast cells cultured in different pH. Confocal microscopy observation of calcofluor white and FITC-antibody double-stained yeast cells. White bars correspond to 10 μm.

An immunofluorescence assay was used to evaluate the immunolocalization of PbSap using *P*. *brasiliensis* yeast cells cultured at pH 6.5 or 4.0 using BSA. We used anti-rPbSap mouse serum in the confocal microscopy experiments and observed its labeling pattern in yeast cells of isolate Pb18 growing at pH 6.5 or 4.0. Fig 4 shows that the yeast cells grown at pH 4.0 (upper panel) and pH 6.5 (middle panel) had similar labeling profiles, with surface fluorescence and intense intracellular labeling, especially for the cells grown at pH 4.0 (**Fig 4**). Control images (lower panel) suggested that the surface labeling was specific for anti-PbSap because the preimmune antibody did not label the yeast cells (**Fig 4**). Collectively, these data suggest that PbSap expression increases at low pH and that acidic pH conditions enhance PbSap activity.

#### Effect of PbSap inhibition in *P*. *brasiliensis* virulence

**Fig 5:**
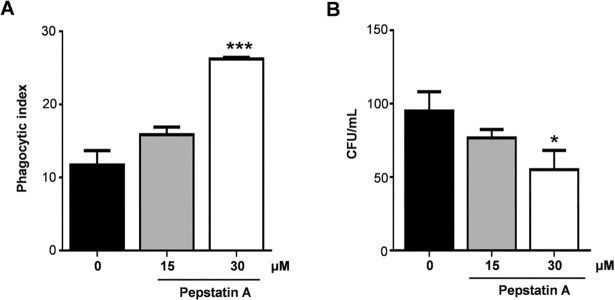
Effect of pepstatin A in interaction assay of *P*. *brasiliensis* and macrophage cells. *P*. *brasiliensis* yeast cells were treated with pepstatin A on concentrations of 15 and 30 μM during 1 h before interaction assay. Pepstatin A increase phagocytosis (A) and enhance antifungal activity of macrophages reducing colony forming units (B). Data were analyzed using Student’s t-test. Error bars correspond to the standard deviation of measurements performed in triplicate, and * indicates a significant difference (*p<0.05).

Secreted aspartyl proteases are sensitive to the inhibitor pepstatin A, a hexapeptide from *Streptomyces* [31]. We observed that macrophages derived from THP1 cells had higher phagocytic indexes (**Fig 5A**) and lower numbers of CFUs (**Fig 5B**) when incubated with *P*. *brasiliensis* yeast cells that had been treated with pepstatin A for 1 h than untreated yeast cells.

**Fig 6:**
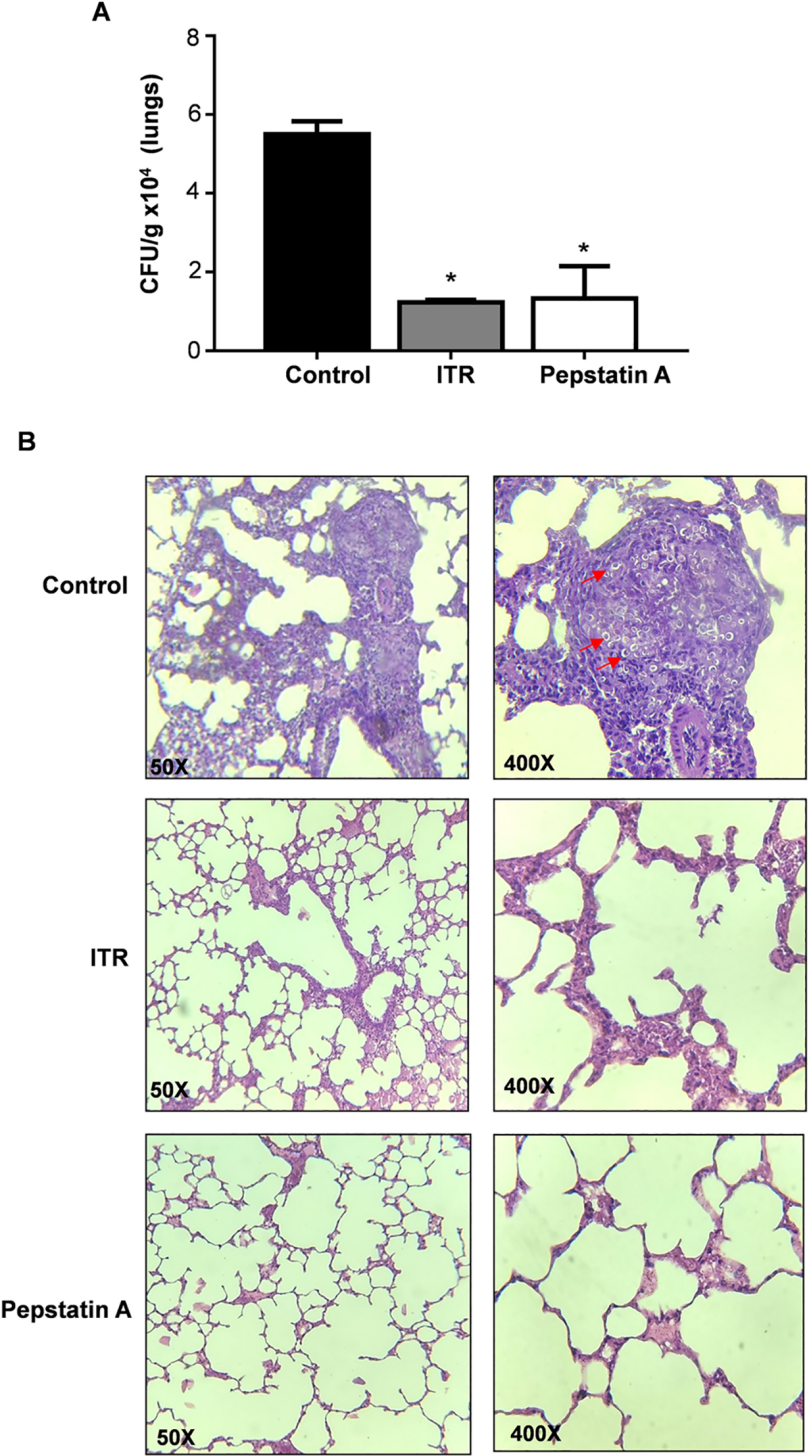
Effect of PbSap inhibition in experimental PCM. Colony-forming units (A) and histological sections of lungs (B) from infected BALB/c mice treated with Pepstain A or Itraconazole (ITR) after 30 days post infection. Groups of mice included only infected (Control) or infected and treated with Pepstatin A or Itraconazole (Treated). The red arrows indicate the location of fungal cells. Data were analyzed using Student’s t-test. Error bars correspond to the standard deviation of measurements performed in triplicate, and asterisks show significant differences (*p*<0.01). H&E staining, x20 magnification.

Protease inhibitors can exert a direct inhibitory effect on *C*. *albicans*, profoundly affecting their growth and pathogenicity [32]. To explore the effects of pepstatin A *in vivo*, we treated BALB/c mice infected with *P*. *brasiliensis* with this aspartyl protease inhibitor. Thirty days after i.t. infection with *P*. *brasiliensis* virulent yeast cells, animals were treated i.p. with pepstatin A for 30 days. Groups of BALB/c mice infected with *P*. *brasiliensis* and treated with ITR were used as positive controls. Untreated infected group were used as controls for experimental PCM. After 30 days, animals treated with inhibitor showed significant reductions in fungal cell numbers in their lungs, as demonstrated by the reduced CFUs/g of tissue recovered from this group compared to that recovered from untreated infected group (*P*<0.05) (**Fig 6A**). The lungs from untreated infected group had multiple pulmonary foci of epithelioid granulomatous inflammation and early pulmonary fibrosis. Mice treated with ITR (positive controls) displayed a similar pattern of fungal burden and organization of the pulmonary parenchyma (**Fig 6A**) as the pepstatin A treated group. Both groups showed few areas with inflammatory infiltrate as well as large areas of preserved lung tissue. These data suggest that PbSap may be a potential virulence regulator since inhibition of PbSap activity affected the course of infection in the experimental PCM model.

### Immunization with PbSap decrease fungal burden in experimental PCM model

**Fig 7:**
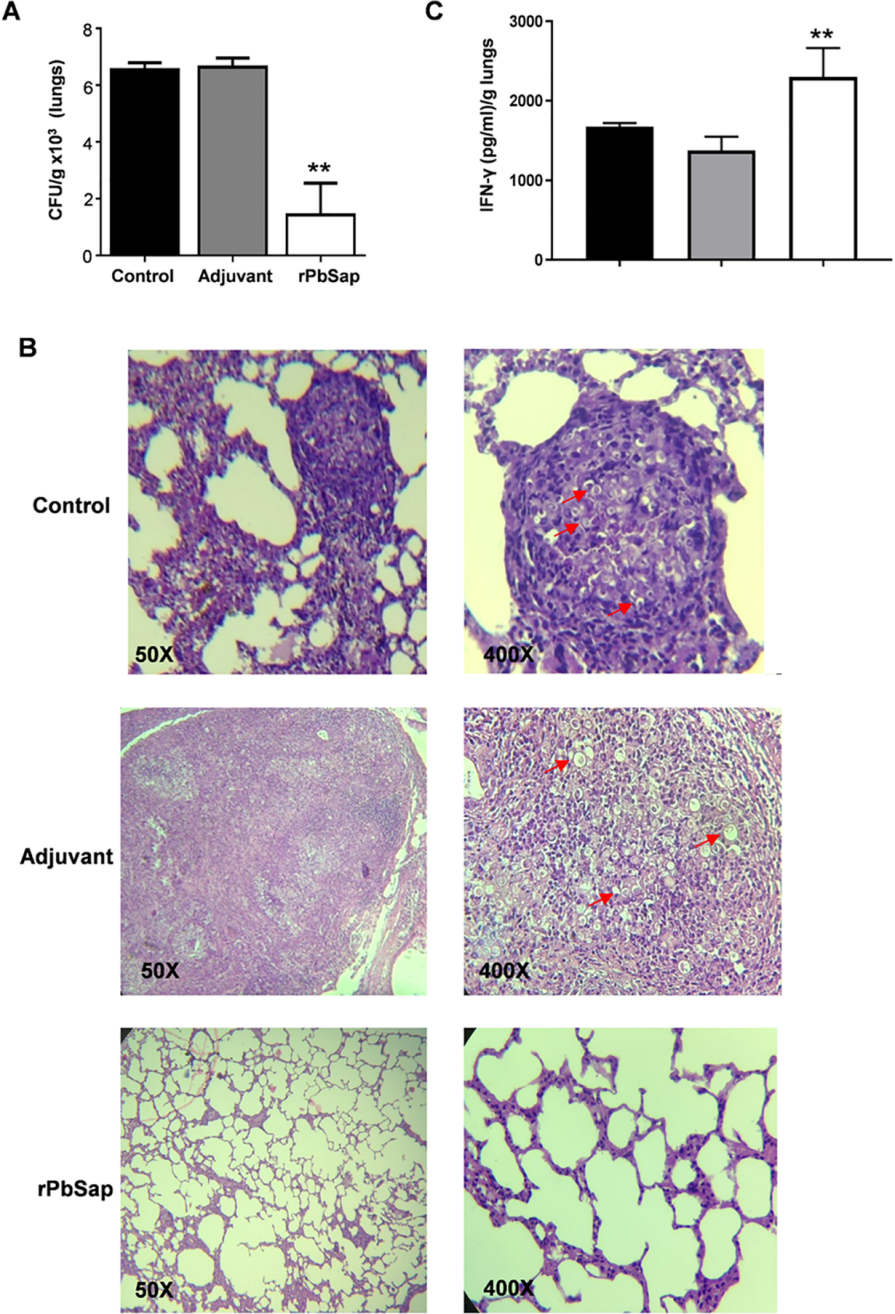
Protection assay by rPbSap during experimental PCM. Colony forming units (A), histological sections of lungs (B) and IFN-γ profile from lung tissue (C) of BALB/c mice challenged i.t. with 1×10^5^ *P*. *brasiliensis* yeast cells after immunization or not with rPbSap. Groups of mice (6 mice) included unimmunized infected (Control), infected and immunized only with adjuvant agent (Adjuvant) and infected and immunized with recombinant protein (rPbSap). The red arrows indicate the location of fungal cells. Data were analyzed using Student’s t-test. Error bars correspond to the standard deviation of measurements performed in triplicate, and **indicates a significant difference (*p*<0.01). H&E staining, x20 magnification.

Groups of BALB/c mice were intraperitoneally (i.p.) immunized with recombinant protein or with adjuvant in three rounds of immunization. The production of a specific antibody response to rPbSap in the immunized mice was confirmed by western blot analysis (**S2 Fig**). After 3 immunizations over 30 days, the BALB/c mice were inoculated intratracheally (i.t.) with yeast cells. The fungal loads in the lungs were evaluated by enumerating the colony forming units (CFUs) after 30 days of infection. The lungs of mice that were immunized with rPbSap had significantly fewer CFUs (1,431 ± 511 CFUs/g of tissue) than the controls (**Fig 7A**): the fungal burdens in the lungs of the mice in the adjuvant and unimmunized groups were 6,636 ± 228 and 6,545 ± 172 CFU/g of tissue, respectively (**Fig 7A**). Lung tissue from mice immunized with rPbSap were stained with H&E and compared to unimmunized infected tissues (**Fig 7B**). As expected, the unimmunized infected lungs showed dense cell infiltrates with high numbers of fungal cells disseminated throughout the lung parenchymas. Similar results were observed in mice treated only with adjuvant. For mice immunized with rPbSap, we observed lung parenchymas that were significantly preserved and lacked fungal cells (**Fig 7B**). IFN-γ production from lung tissue was assessed from controls and BALB/c mice immunized with rPbSap. Our results showed significant increase in IFN-γ production in the lung cells of immunized group compared with controls (**Fig 7C**). Taken together, our results showed that immunization with rPbSap prevent the aggravation of *P*. *brasiliensis* infection.

## Discussion

Given the increasing worldwide incidence of fungal infections, characterizing new virulence factors is very important for understanding the pathogenicity of fungi. In *Candida* spp., Sap is the most important virulence factor [4]. This protein family is encoded by at least 10 *SAP* genes (*SAP1*–*SAP10*) [4,33], which are differentially expressed during distinct patterns of infection [33,34].

In a previous report, we compared the proteomes of virulent and attenuated *P*. *brasiliensis*; several proteins that have been described as virulence regulators in other fungi were up-regulated in the virulent Pb18 strain [15]. Vacuolar protease A (PADG_00634), also named aspartyl protease, was one of the up-regulated proteins in virulent *P*. *brasiliensis*. Transcriptome analysis of the M-Y transition in the Pb01 strain revealed that aspartyl protease transcript levels were up-regulated [13]. The same profile was observed in Pb18; transcript levels of *PbSAP* increased during an early stage of the M-Y transition and remained elevated until it completed. In contrast, during the Y-M transition, we observed a decrease in the transcript levels of *PbSAP*. Temperature changes represent a stressor, and in Pb18, the yeast cells respond to this stress by increasing their transcript levels of *PbSAP*. Dimorphic switching appears to be intimately linked to pathogenicity. Previous studies have reported the up-regulation of genes involved in diverse cellular pathways during the M-Y transition in *P*. *brasiliensis*, including genes encoding putative virulence factors as heat shock proteins HSP70, HSP82 and HSP104 [3,30]. These heat shock have similar patterns of expression when compared with the transcript levels of *PbSAP* in the dimorphic transition. In addition, we observed several putative ncHSE motifs in the *PbSAP* promoter. Conventional HSE motifs are typically composed of three to six continuous and/or inverted pentameric units of nGAAn [35]; in ncHSE, the gaps between the repetitive units can vary [36]. Discontinuous HSEs, which are ncHSEs, are found in approximately half of the yeast heat-shock factor (yHSF) target genes [18,36–38]. Thus, these data suggest that PbSap could participate in the temperature change response of *P*. *brasiliensis* and consequently contribute to its adaptation to the host environment.

To assess the PbSap antigenicity profile, we produced it as a 44-kDa recombinant protein and incubated it with sera from patients diagnosed with PCM, which efficiently recognized rPbSap in an immunoblotting assay. On the other hand, the rPbSap was not recognized by sera from patients with candidiasis, histoplasmosis and aspergillosis. Serological analysis is the main diagnostic indicator of PCM [39], and the characterization of new antigens can improve the serodiagnosis of PCM. During infection, *P*. *brasiliensis* antigens activate B lymphocyte cells, which produce immunoglobulins (Igs) that play a role in host defense against a variety of pathogens. High concentrations of Igs have been found in the bodily fluids of PCM patients [40]. Anti-Sap antibodies were observed in sera from patients with candidemia, indicating the presence of Sap antigens during systemic infection [41]. However, further studies are necessary to confirm the potential of PbSap for PCM diagnosis.

In this study, we report the efficacy of rPbSap immunization. The data obtained showed that rPbSap immunization decreased fungal burden in an experimental PCM model. Saps from *C*. *albicans* are known to be immunogenic and to strongly induce a host protective response in animal models [8]. The identification of *P*. *brasiliensis* antigens and the evaluation of their role in parasite-host interactions are fundamental for the study of immunization procedures, which may lead to the prevention of infection. Some immunization experiments using *P*. *brasiliensis* recombinant proteins have already been performed. Morais and co-workers [42] conducted immunization procedures using rPb27 and showed that the rPb27-immunized group had lower levels of lung fibrosis, demonstrating the protective effect of rPb27. Assis-Marques and co-workers [43] conducted similar studies using rGp43 and observed that immunized animals had less frequent, more compact granulomas with fewer fungal cells as well as fewer fungal cells in the lungs and spleen than non-infected animals. In addition, the organs of immunized animals showed high levels of interleukin (IL)-12 and interferon (IFN)-γ, suggesting a protective T helper 1 (Th1) response. Muñoz and co-workers [44] demonstrated that immunization with P10, a peptide from the Gp43 antigen, promotes a specific immune response even in immunocompromised BALB/c mice that is able to confer protection against *P*. *brasiliensis*. The protection conferred by immunization with rPbSap suggests that the antigen appears to be a promising candidate against *P*. *brasiliensis* infection. However, additional characterization can contribute to better evaluate and to compare PbSap protection with other antigens such as, rPb27, rGp43 and P10. Antisense RNA technology and *Agrobacterium tumefaciens*-mediated transformation have confirmed the importance of rPb27 and rGp43 in *P*. *brasiliensis* virulence [45,46].

To understand the role of PbSap in experimental PCM we inhibited enzymatic activity using a pharmacological inhibitor (pepstatin A, an inhibitor of aspartyl protease). Our results suggest that PbSap is a potential virulence regulator, considering that inhibition of PbSap using pepstatin A significantly reduced experimental PCM infection. Pepstatin A has also shown to be a potent prophylactic agent for the prevention of lethal *C*. *albicans* infections [6]. HIV protease inhibitors that are used in the treatment of HIV disease efficiently reduce candidiasis by inhibiting Sap activity [47–50]. Indinavir affects the virulence of *Cryptococcus* neoformans [51] by reducing its protease activity and capsule production [52]. Saquinavir or ritonavir synergistically interacts with itraconazole against *H*. *capsulatum* [53]. The inhibition of PbSap represents a promising strategy for the design of new drugs, and a combined therapy using anti-fungal drugs and Sap inhibitors could increase the efficiency of treatment for *P*. *brasiliensis* infection.

PCM is considered a classic granulomatous disease and macrophages play an important role in defense against the disease [3]. *P*. *brasiliensis* is a facultative intracellular pathogen that can survive inside macrophages, and this behavior is likely an important factor in its pathogenicity [27,54]. Using a proteomics approach, several proteins that were up-regulated in *P*. *brasiliensis* yeast cells during their interaction with macrophages have been described as virulence factors [27]. The result of this interaction is the phagocytosis of *P*. *brasiliensis* by macrophages [3], which is followed by fusion of the microorganism-containing phagosomes with cellular lysosomes to form phagolysosomes. The low pH (4.7–4.8) inside the phagolysosomes supports the activity of the host’s acid lysosomal enzymes and is an essential host strategy for killing most pathogens [54,55]. However, the low pH is also optimal for the enzymatic activity of aspartyl proteinase [4,31]_._ After their ingestion by phagocytic cells, *C*. *albicans* and *C*. *tropicalis* were shown to express Sap antigens [56], suggesting a role for Sap in fungal adaptation to the intracellular environment. Our data demonstrated that at low pH, the expression of PbSap increased, while only low levels of expression were observed above pH 6.5. In mammalian hosts, few environments have a low pH. The expression of PbSap is possibly enhanced by phagosome–lysosome fusion due to the pH change that occurs. As reported previously, the expression of Sap4, Sap5 and Sap6 increased in *C*. *albicans* during its interaction with macrophages [57]. Phagosome-lysosome fusion intensifies the activity of potential pathogenic factors, which benefits microbial intracellular survival, as observed for *Trypanosoma cruzi* and *Mycobacterium tuberculosis* [58]. In *C*. *neoformans* the major aspartyl peptidase 1 (May1) showed relevant importance for survival in acidic host environments such as macrophage phagolysosomes. In a mouse model may1Δ strains displayed attenuated profile [59].

Collectively, our results suggest that PbSap plays a role in the adaptation of *P*. *brasiliensis* to the defense systems of its hosts. While it is not requisite for fungal survival, PbSap is an essential element for fungal infection, acting as a potential virulence regulator. In addition, the inhibition of PbSap ameliorates *P*. *brasiliensis* infection and its progression, demonstrating that this protein can be used as a molecular target for the development of new strategies and therapies for PCM and other fungi. Additional studies to evaluate the immune response and long-term infection in immunized mice can contribute to better understand the role of PbSap in the immune response and its contribution to fungal-host interaction. In addition, studies using rPbSap for the serodiagnosis of PCM could also help to improve and standardize immunological diagnoses as well as patient follow-up. This approach is under investigation in our laboratory.

## Supporting information

S1 Fig Characterization of PbSap glycosylation of *P*. *brasiliensis*. Protein extracts from Pb18 were treated with endoglycosidase H and followed by immunoblot analysis. Treatment with endoglycosidase H produced a protein species of 44 kDa (red arrow).
(TIF)

S2 Fig Antibody anti-rPbSap production. Immunobloting analysis of control, adjuvant and rPbSap mouse immune sera showing the effective production of anti-rPbSap.
(TIF)
