# Supplementary figures and images for: Secreted aspartyl proteinase (PbSap) contributes to the virulence of Paracoccidioides brasiliensis infection
Source: PLoS Negl Trop Dis. 2018 Sep 27;12(9):e0006806. doi: 10.1371/journal.pntd.0006806 (PMC6177206; doi:10.1371/journal.pntd.0006806)

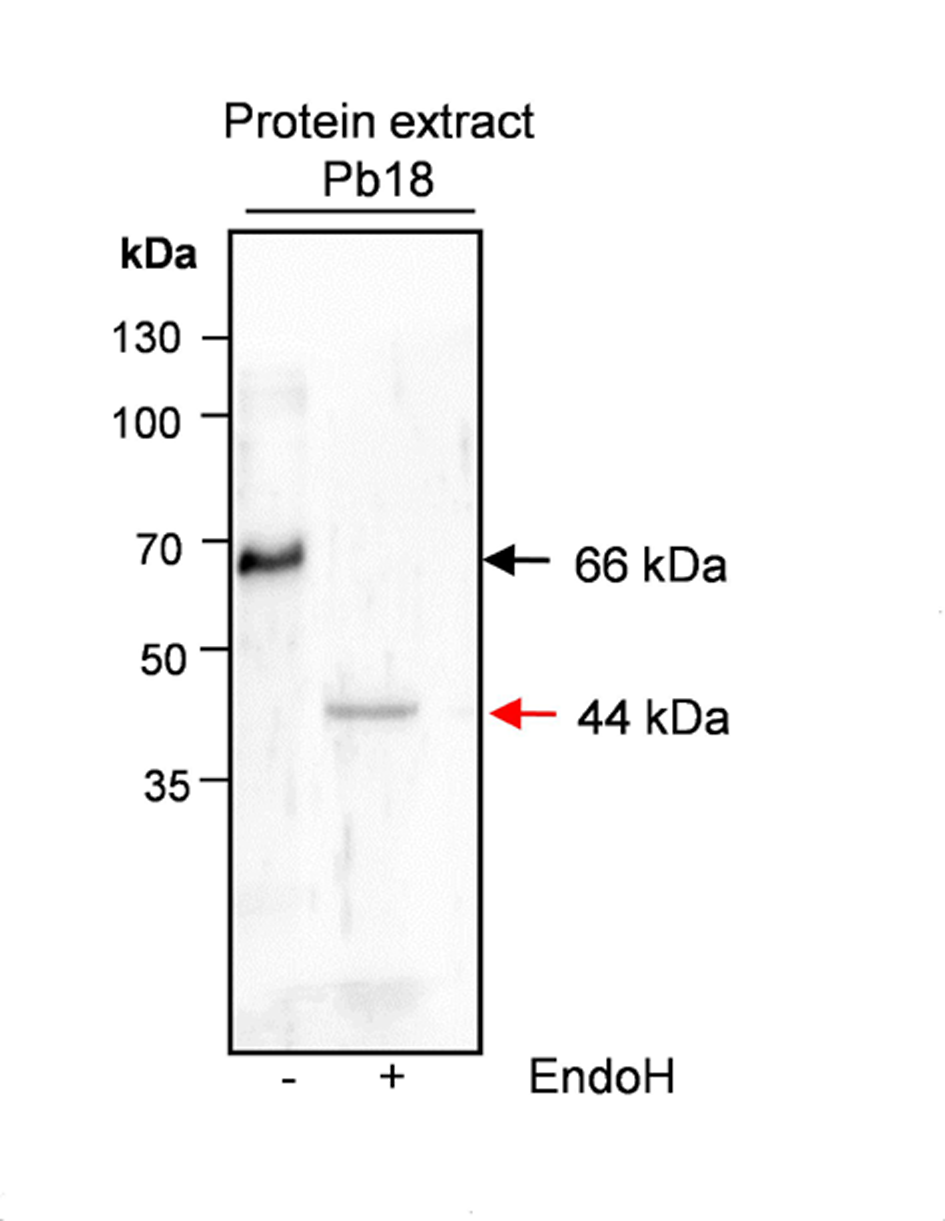

Supplement: S1 Fig — Protein extracts from Pb18 were treated with endoglycosidase H and followed by immunoblot analysis. Treatment with endoglycosidase H produced a protein species of 44 kDa (red arrow). (TIF) [file pntd.0006806.s001.tif]

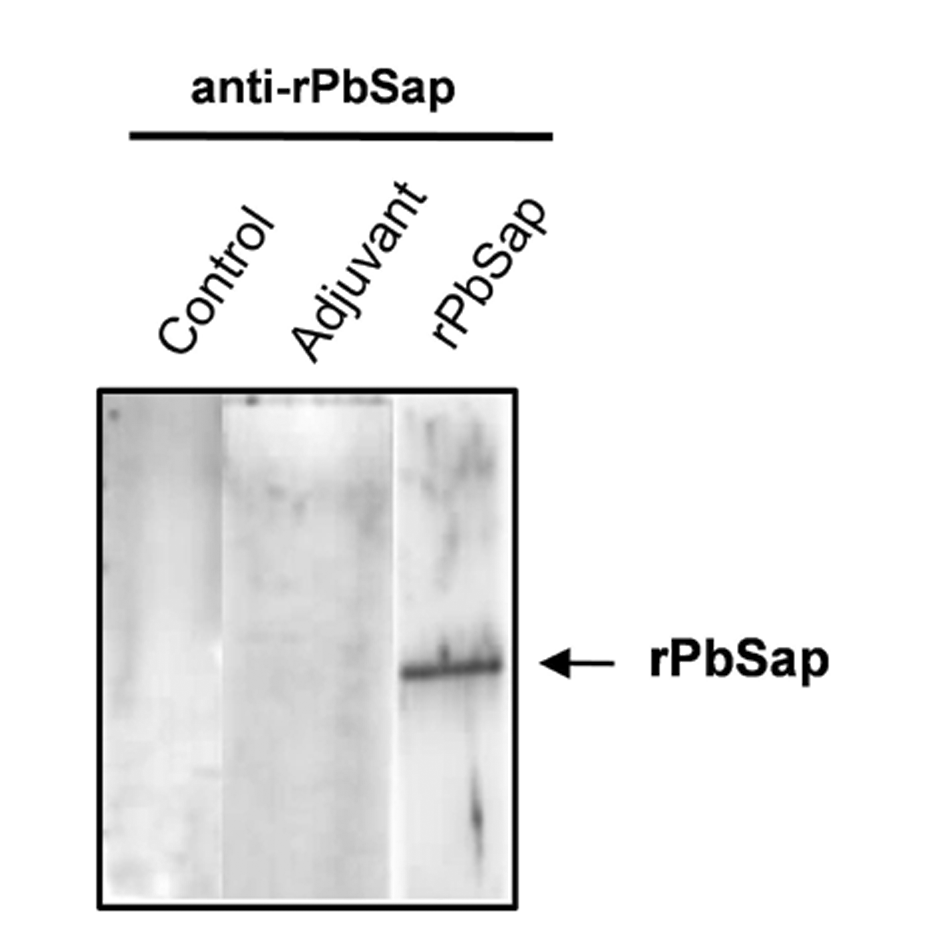

Supplement: S2 Fig — Immunobloting analysis of control, adjuvant and rPbSap mouse immune sera showing the effective production of anti-rPbSap. (TIF) [file pntd.0006806.s002.tif]
